# Sensitive Detection of Norovirus Using Phage Nanoparticle Reporters in Lateral-Flow Assay

**DOI:** 10.1371/journal.pone.0126571

**Published:** 2015-05-15

**Authors:** Anna E. V. Hagström, Gavin Garvey, Andrew S. Paterson, Sagar Dhamane, Meena Adhikari, Mary K. Estes, Ulrich Strych, Katerina Kourentzi, Robert L. Atmar, Richard C. Willson

**Affiliations:** 1 Department of Chemical and Biomolecular Engineering, University of Houston, Houston, Texas, United States of America; 2 Department of Biology and Biochemistry, University of Houston, Houston, Texas, United States of America; 3 Department of Molecular Virology and Microbiology, Baylor College of Medicine, Houston, Texas, United States of America; 4 Department of Medicine, Baylor College of Medicine, Houston, Texas, United States of America; 5 Houston Methodist Research Institute, Houston, Texas, United States of America; 6 Tecnológico de Monterrey, Departamento de Biotecnología e Ingeniería de Alimentos, Centro de Biotecnología FEMSA, Monterrey, Nuevo León, Mexico; George Mason University, UNITED STATES

## Abstract

Noroviruses are recognized worldwide as the principal cause of acute, non-bacterial gastroenteritis, resulting in 19-21 million cases of disease every year in the United States. Noroviruses have a very low infectious dose, a short incubation period, high resistance to traditional disinfection techniques and multiple modes of transmission, making early, point-of-care detection essential for controlling the spread of the disease. The traditional diagnostic tools, electron microscopy, RT-PCR and ELISA require sophisticated and expensive instrumentation, and are considered too laborious and slow to be useful during severe outbreaks. In this paper we describe the development of a new, rapid and sensitive lateral-flow assay using labeled phage particles for the detection of the prototypical norovirus GI.1 (Norwalk), with a limit of detection of 10^7^ virus-like particles per mL, one hundred-fold lower than a conventional gold nanoparticle lateral-flow assay using the same antibody pair.

## Introduction

Noroviruses are RNA viruses belonging to the *Caliciviridae* family (with Norwalk virus being the type species of the genus), and are responsible for most outbreaks of gastrointestinal infection reported in the popular press [1–3]. Outbreaks often occur in close-contact settings such as cruise ships, military vessels and environments, hospitals, nursing homes, and schools. Noroviruses were found to be the leading cause of hospital infection outbreaks and accounted for the most department closures in U.S. hospitals from 2008 to 2009 [4] and were the single most important cause of disease-outbreak-related morbidity aboard ships in the U.S. Navy [5, 6]. In total, noroviruses are estimated to cause 19–21 million illnesses per year in the U.S., with 56,000–71,000 hospitalizations and 570–800 deaths [3]. The transmission route is most often person-to-person (fecal-oral mode or through inhalation of airborne droplets of vomitus) or food-borne, originating from food handlers [7]. Noroviruses have a high infectivity; the 50% human infectious dose is estimated to be 1,015–1,320 virions [8, 9]. Asymptomatic individuals, as well as those who have recovered from symptoms, can shed virus particles for three weeks or longer after exposure [10, 11]. Noroviruses are also more resistant to disinfection techniques than most bacteria and other viral pathogens [12]. In the midst of an outbreak there is a need to quickly identify the cause of the symptoms in order to determine the precautions needed, e.g. antibiotics or implementation of containment [6] and to limit the outbreak duration [13] which is especially critical in closed environments such as cruise ships or military settings.

The traditional diagnostic tools, electron microscopy, RT-PCR, ELISA and various recently reported improved and combined versions of these (e.g. [14–17]), require sophisticated and expensive instrumentation, and are considered too laborious and slow to be useful during severe outbreaks. Point-of-care detection methods, like the well-established immunochromatographic lateral-flow assays (LFAs), would be useful in non-hospital settings where these outbreaks often occur and for screening food handlers. Several gold nanoparticle-based immunochromatographic tests for the detection of noroviruses have been reported [18–22]. The most studied test is the RIDAQUICK rapid test developed by R-Biopharm though mainly used as a yes/no assay with no limit of detection (LoD) reported. RIDAQUICK is a qualitative, immunochromatographic assay for determining the presence of genogroups 1(GI) and 2 (GII) noroviruses in stool samples with a reported clinical sensitivity of 92% (manufacturer literature). The assay employs both biotinylated anti-norovirus antibodies and gold-labeled anti-norovirus antibodies; when target noroviruses are present in the sample, virions associate with the antibodies while flowing through the strip. A streptavidin test line captures the gold-labeled migrating complexes via the biotinylated anti-norovirus antibodies. Migrating gold-labeled antibodies not bound in the complex are bound later at the control line. The main drawback for these traditional LFAs using colored particles such as blue latex or gold nanoparticles, is the high LoD [23]. It is evident from the great commercial and academic interest in developing alternative LFA reporters and reader technologies that there is a felt need for more sensitive rapid tests.

Several efforts have been reported to improve the analytical sensitivity in LFAs, including pre-concentration [24, 25] or the use of enzymes on the reporter particles (typically giving a ten-fold decrease in LoD [26–29]). Photoluminescent particles have also been used to decrease the LoD of LFAs by 10 to 100-fold compared to gold nanoparticle LFA, but require complex instrumentation [30–32]. Our previous work established that phage LFAs are inherently much more sensitive (achieving as much as 1000-fold lower LoD) than gold nanoparticle LFAs that employ the same antibody pair [33]).

This study was undertaken to extend the use of our previously-developed excellent phage LFA reporters to a practical diagnostic need. We used ELISA to identify an optimized antibody sandwich pair for the detection of non-infectious virus-like particles (VLPs) from GI.1 Norwalk (the first-recognized norovirus, considered to be the prototype virus for the genus [34, 35]). Thereafter, the utility of this antibody sandwich pair was confirmed in both bacteriophage and gold nanoparticle LFA. The LoD was improved 100-fold using bacteriophage nanoparticles as reporters compared to the conventional gold nanoparticle LFA.

## Materials and Methods

### Materials

SAM-AviTag M13 phage were the generous gift of Dr. Brian Kay, UIC (Chicago, IL). Tetracycline was purchased from Teknova (Hollister, CA). Biotin ligase kit (BirA500) was purchased from Avidity (Aurora, CO). EZ-Link Sulfo-NHS-LC-Biotin (21335), NeutrAvidin (31000), 1-step ultra TMB-Blotting solution (37574), 1-Step Ultra TMB-ELISA solution (34028), phosphate buffer saline (PBS) tablets (pH 7.4) (IC-N2810307), Nunc Medisorp 96-well plates and Pierce Reacti-bind 96-well plates, Neutravidin (15128) were purchased from Thermo Scientific (Rockford, IL). Float-a-lyzers (100 kDa and 300 kDa) were purchased from Spectrum Laboratories (Rancho Dominguez, CA). 2x Brilliant III Ultra-Fast SYBR Green PCR Master Mix was purchased from Agilent (Santa Clara, CA). Anti-norovirus antibodies (10–1510 and 10–1511, called F1 and F2 respectively, below) were purchased from Fitzgerald (Acton, MA), NV3901 [36] and NV23 [37] were obtained from cultivation of hybridoma cells. Lateral-flow assay nitrocellulose membranes (FF80HP), sample pads (Fusion 5) and absorbent materials (CF5) were all purchased from GE Healthcare (Piscataway, NJ). Anti-M13 antibodies (NB100-1633) were purchased from Novus Biologicals (Littleton, CO) and HRP/anti-M13 monoclonal conjugate (27-9421-01) were purchased from GE Healthcare Life Sciences (Pittsburgh, PA). Streptavidin-HRP (S5512), 3350 g/mol polyethylene glycol (PEG, P3640), Triton X-100 (X100), Tween 20 (P9416) and bovine serum albumin, BSA, (A7906) were purchased from Sigma Aldrich (St. Louis, MO). PVC backing cards (MIBA-020) and gold nanoparticles (40 nm, OD 1, 10^11^/mL, CG-020) were purchased from DCN Diagnostics (Carlsbad, CA).

### Norwalk VLP production

Norwalk VLPs were expressed and purified as reported previously [38]. Briefly, the major capsid proteins (VP1 and VP2) were expressed, from a baculovirus vector, in Sf9 insect cells. The VLPs were purified using a cesium chloride gradient, and the structure was confirmed by electron microscopy. VLPs were stored in PBS at 4°C.

### Culture and titration of M13 bacteriophage

SAM-AviTag M13 phage were grown and titered as previously described [39]. Briefly, *E*. *coli* ER2738 were grown in 5 mL LB media with tetracycline (15 μg/mL) to mid-log phase and shaking at 37°C. The bacteria were then infected with 5 μL phage stock (~10^12^ pfu/ml) and grown for 2 h at 7°C with shaking. This pre-culture was then transferred to 500 mL 2xTY medium and incubated overnight at 37°C on a shaker. Bacteria were separated from phage in the supernatant by centrifugation (30 min, 4,000 x g). Phage were then precipitated, twice, using a PEG solution containing 20% PEG3350 and 2.5 M NaCl as previously described [39]. PBS containing 0.02% sodium azide was added to the final phage pellet. The phage solution was kept on ice for 10 min and thereafter centrifuged (10 min, 14,000 x g) to eliminate any remaining bacteria. The concentrated phage stock was stored in PBS, 0.02% sodium azide, at 4°C. Phage titers were determined on X-Gal/IPTG plates as described previously [40]. Absorbance at 260 nm was measured to estimate the phage titer using an empirical formula derived from the titered phage stock (phage/mL = 6x10^11^xOD260) [41]. Additionally, PCR of the phage DNA was used to estimate phage concentration and recovery during and after phage modification against a standard curve derived from a series of dilutions of (unmodified) phage that had been titered on X-Gal/IPTG plates. PCR was performed in a MX3005P QPCR System (Agilent Technologies) by mixing 5 μL of diluted phage with 15 μL PCR master mix (0.1 μL of 10 μM forward primer, 0.1 μL of 10 μM reverse primer, 10 μL of 2xPCR mix and 4.8 μL of RNase- and DNase-free DI water) to achieve 20 μL total PCR volume. The Ct value versus number of phage particles standard curve was derived from a 10-fold dilution series of a titered stock. The AviTag-targeted PCR primers, designed in-house, were as follows: Forward: 5’-GTTGTTTCTTTCTATTCTCACTCC-3’, and Reverse: 5’-CAGACGTTAGTAAATGAATTTTCTG-3’. The PCR conditions were: 10 min at 95°C, 40 cycles of 30 sec at 95°C, 30 sec at 62°C and 30 sec at 72°C, followed by dissociation step (1 min at 95°C, 30 sec at 55°C, and 30 sec at 95°C).

### Bacteriophage M13 functionalization

The AviTag peptides displayed on the phage protein III were enzymatically biotinylated using *E*. *coli* biotin ligase according to the manufacturer’s instructions and the phage was purified using PEG precipitation, as described above. The biotinylated AviTag phage were incubated with a 100-fold excess of NeutrAvidin and then purified by a Spin-Dialyzer (100 kDa cutoff). Bovine serum albumin (BSA) and monoclonal anti-norovirus (Fitzgerald 10–1510, F1) antibody were biotinylated using EZ-Link Sulfo-NHS-LC-Biotin reagent using a 20-fold molar excess of biotin reagent according to the manufacturer’s instructions. NeutrAvidin-functionalized phage were incubated with a 10-fold molar excess of biotinylated antibody for 1 h at room temperature, before uncoupled antibodies were removed using a 300 kDa Float-a-lyzer. The antibody-phage-conjugate was quantified using absorbance measurements at 260 nm and PCR, and thereafter stored in PBS at 4°C.

### ELISA

To select the proper antibody pair to be used for the LFA, various antibody combinations were evaluated through ELISA, both as capture and as detection (biotinylated for coupling to phage reporter particles) agents. Nunc MediSorp plates were used to adsorb the capture antibodies (2 μg/mL in PBS), overnight at room temperature (RT). Liquid was aspirated and the wells were blocked with 2% BSA solution (in PBS) for 1 h at RT on a shaker. Then, dilutions of Norwalk VLPs in dilution buffer (PBS containing 1% BSA) were captured for 2 h, shaking at RT. After washing three times with wash buffer (PBS containing 0.1% Tween 20) using a Tecan Hydroflex plate washer, biotinylated detection antibody was added to each well (10 ng/well in incubation buffer). The unbound detection antibody was removed by washing three times with wash buffer, and then streptavidin-HRP (0.2 μg/mL, 100 μL/well) was added and incubated for 30 min while shaking at room temperature. Excess conjugate was removed by washing three times with wash buffer and then 1-step Ultra TMB ELISA solution (100 μL) was added. After 20 min, 100 μL 2M H_2_SO_4_ was added to stop the reaction. Absorbance was measured at 450 nm using a Tecan Infinite M200 Pro Plate reader. The screening ELISA was performed twice, each time in duplicate, for the range of VLP concentrations shown.

### Gold nanoparticle functionalization

Gold nanoparticles were functionalized by adding 100 μl of 4 mM K_2_CO_3_ to 1mL of the stock particles. Then, 10 μg monoclonal anti-norovirus antibody (Fitzgerald 10–1510, F1) were added for 20 min at 25°C on a rotator. BSA, 100 μL (10% (w/v)) was added to block the nanoparticles. After 20 min, the functionalized nanoparticles were collected by centrifugation (5 min, 10,000 x g). The particles were washed once with 1 mL of storage solution (PBS, pH 7.4, 1% (w/v) BSA, 10% (w/v) sucrose), then suspended in 100 μL of storage solution and stored at 4°C. To estimate the concentration of gold nanoparticles, their absorbance at 520 nm was measured and compared to the stock (particles/mL = 10^11^xOD520).

### Preparation of LFA strips

To form test and control lines, antibodies were spotted onto nitrocellulose (FF80HP) using a Lateral-Flow Reagent Dispenser (Claremont BioSolutions, Upland, CA) equipped with an external syringe pump (Chemyx, Stafford, TX). Anti-norovirus antibodies (Fitzgerald, 10–1511, F2, in 10 mM phosphate buffer, pH 6.9) were used at a line concentration of 1 μg/cm. For the control lines in phage LFA, anti-M13 antibodies were dispensed at a line concentration of 0.25 μg/cm and for the gold nanoparticle LFA, anti-mouse antibodies were deposited at a line concentration of 0.2 μg/cm. The membranes were dried at 37°C for 1 h and then stored, desiccated at room temperature, for at least 20 h before use.

The nitrocellulose membrane (length 40 mm) was adhered to a backing card, and Fusion 5 membrane (length 20 mm) was applied as a sample pad. CF 5 was used as the absorbent pad. The membrane was then cut into 5 mm wide strips using a paper cutter and the strips were stored with desiccant at room temperature until used.

### Lateral-flow assay

**Fig 1:**
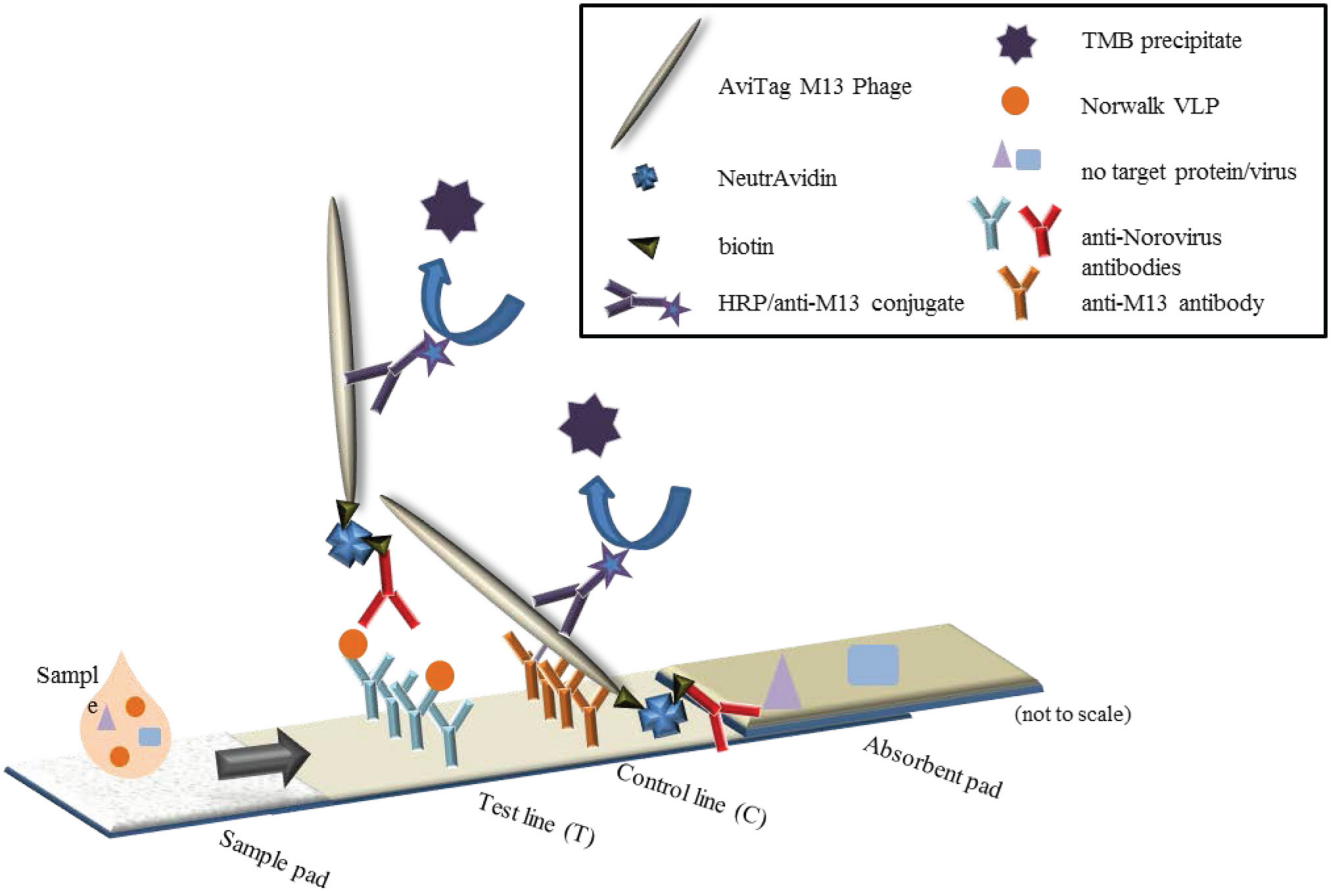
Phage lateral-flow assay detecting Norwalk VLPs. Assay membrane is nitrocellulose (FF80HP, 5x40 mm), sample pad is Fusion 5 (5x20 mm), Absorbent pad is CF5 (10x30 mm). Control line consists of anti-M13 antibodies (0.25 μg/cm) and test line is anti-Norwalk monoclonal antibodies (1.0 μg/cm).

The assay configuration is illustrated in Fig 1. Norwalk VLPs were diluted in PBS containing 2% BSA, and a 100 μL sample was pipetted onto the sample pad. Then 50 μL wash buffer (PBS, 1% Tween 20, 1% Triton X-100, 0.5% PEG3350) was added onto the sample pad, followed by 5*10^9^ phage construct particles (10 μL in PBS, 2% BSA) and another 50 μL of wash buffer. Subsequently, 10 μL anti-M13 HRP conjugate was added and the excess was removed with 120 μL wash buffer. Finally, 30 μL TMB Blotting solution was added all over the strip. At defined time intervals, scans were taken to record the developed colorimetric signal using a Perfection V600 flatbed color scanner (Epson, Long Beach, CA). The scanned images were analyzed using ImageJ’s Gel Analysis Tool [42], plotting the line intensity profile and calculating the total area of each peak. The intensity of the test line was divided by the sum of the test line and the control line. A standard approach was used to estimate the LOD as the lowest analyte concentration that gave a signal (S_t_) clearly distinguishable from the no-target sample signal (S_t_ > S_no-target_ + 3SD_no-target_). Comparison of the no-target control to positive LFAs using the t-test was also performed (S1 Table).

## Results and Discussion

### Screening of antibody pairs

**Fig 2:**
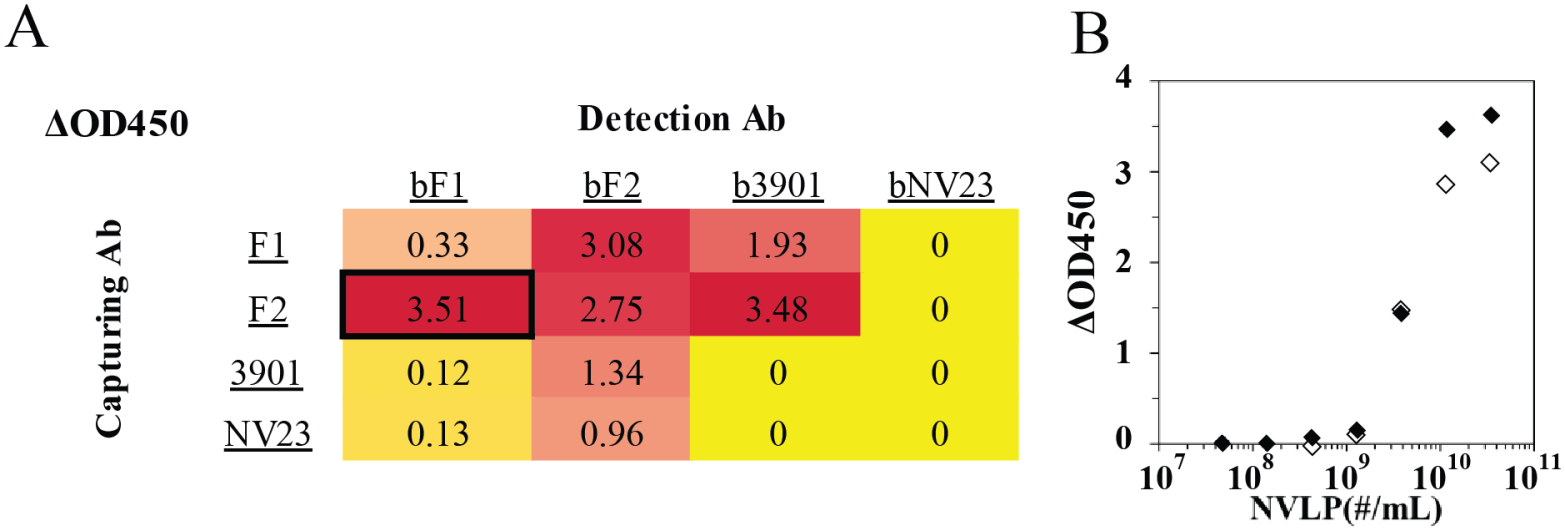
Detection of Norwalk VLPs using a sandwich ELISA. A) Antibody pair screening for the detection of Norwalk VLPs; values correspond to the absorbance for a sample for 10^9^ VLPs offered; background absorbance for no VLP sample was subtracted (typical value ~0.1). Red color denotes maximum ΔOD450 observed in the ELISA, yellow lowest, and a smooth color gradient in between. Black box denotes the sandwich pair that was used in LFA. B) Sandwich ELISA detecting Norwalk VLPs where F2 was used as the capturing antibody. For the detection biotinylated F1 and streptavidin HRP (antibody sandwich, closed symbols), or the phage construct (Antibody-NeutrAvidin-AviTag phage) and anti-M13/ HRP conjugate (phage sandwich; open symbols) were used.

The performance of immunoassays depends critically upon the use of the optimal antibody sandwich pair with a specific orientation [43]. A range of antibodies were initially checked, both in native and biotinylated form, to confirm binding to our VLPs. Thereafter, all binding antibodies were evaluated in all pairwise combinations in a sandwich ELISA. Results of the evaluation of all combinations and orientations can be seen in Fig 2A, where differences in absorbance at 450 nm (ΔOD450), for a sample containing 10^10^ VLP/mL and a sample with no VLPs (typical value ~0.1), for several pairs of antibodies are given. The antibody pair chosen for further study of the detection of norovirus GI.1 Norwalk VLPs was the F2 (capturing) + F1 (biotinylated; detection) antibodies from Fitzgerald. This pair was chosen from among the best-performing candidates because it is commercially available and recommended by the supplier. This pair was further evaluated using a series of VLP concentrations in a sandwich ELISA using F2 antibody as the capturing agent and either F1 biotinylated antibody (closed symbols) or the F1 antibody-NeutrAvidin-phage construct (open symbols) for detection (Fig 2B).

### Antibody phage construct

After phage preparation, the AviTag peptide expressed on one of the minor coat proteins, pIII, was biotinylated using biotin ligase and NeutrAvidin was then bound to the biotinylated AviTag. These phage constructs were evaluated using ELISA on NeutrAvidin plates and Nunc Medisorp plates with biotinylated BSA adsorbed onto them, to confirm proper functionalization of the phage (data not shown). During the preparation of the phage construct, the phage titer was determined by PCR with comparison to a standard curve showing the dependence of Ct value on phage concentration. Finally, biotinylated anti-Norwalk antibodies were conjugated to the NeutrAvidin-phage.

### Lateral-flow assay

**Fig 3:**
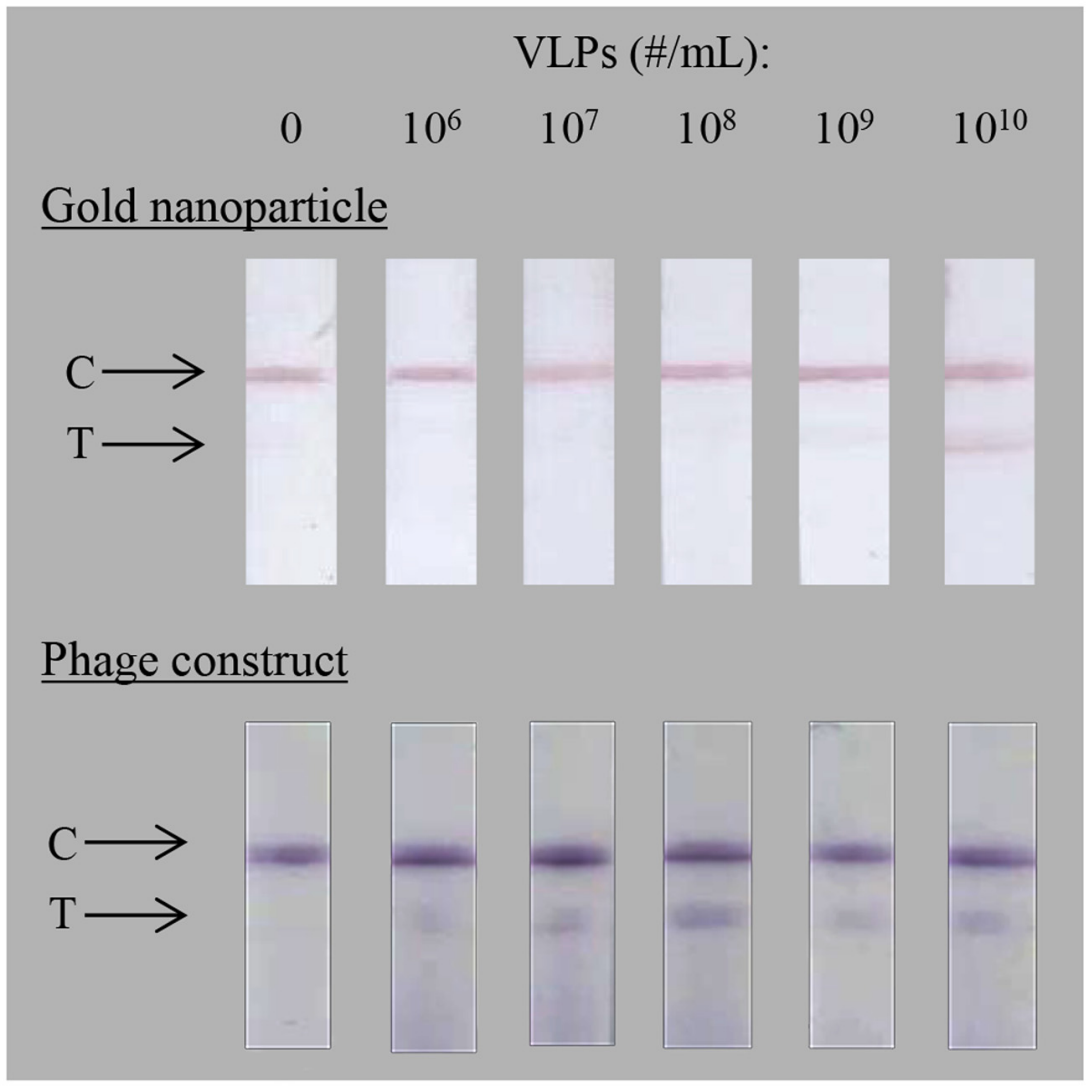
Detection of Norwalk VLPs in lateral-flow assay (LFA). Norwalk VLPs in 100 μL are detected using anti-Norwalk antibodies in the test line (T); gold nanoparticle (top row) and antibody-phage construct followed by HRP/anti-M13 conjugate (bottom row). Control line (C) consists of anti-mouse antibodies for the gold nanoparticle LFA and anti-M13 antibodies for the phage LFA. Nitrocellulose FF80HP was used as test membrane, Fusion 5 as sample pad and CF5 as absorbent pad. All images were equally gamma-corrected (gamma-correction factor = 0.45) to compensate for contrast lost in the overexposed, scanned images and better represent the naked-eye appearance of the raw strips.

LFA for the detection of GI.1 Norwalk VLPs was demonstrated in this study. In Fig 3 representative gold nanoparticle LFA (top) and phage construct LFA (bottom) strips for a dilution series of Norwalk VLPs are compared. The top line on each strip is the control line, used to confirm correct flow of liquid and detection agent along the membrane. The lower line is the test line used for the detection of the Norwalk VLPs. The assay specificity was tested using 10^9^ MS2 virus particles instead of VLPs, and also with NeutrAvidin phage with no antibody attached, on captured VLPs. Those negative controls showed no background signal and the strips were indistinguishable from the zero VLPs samples (data not shown). There were some variations in intensity between strips to which the same concentration of VLPs had been added. Assay reproducibility could be improved with better consistency of LFA strip production, for example cutting, line dispensing, assembly of membranes, and/or in reagent delivery, etc. [23].

**Fig 4:**
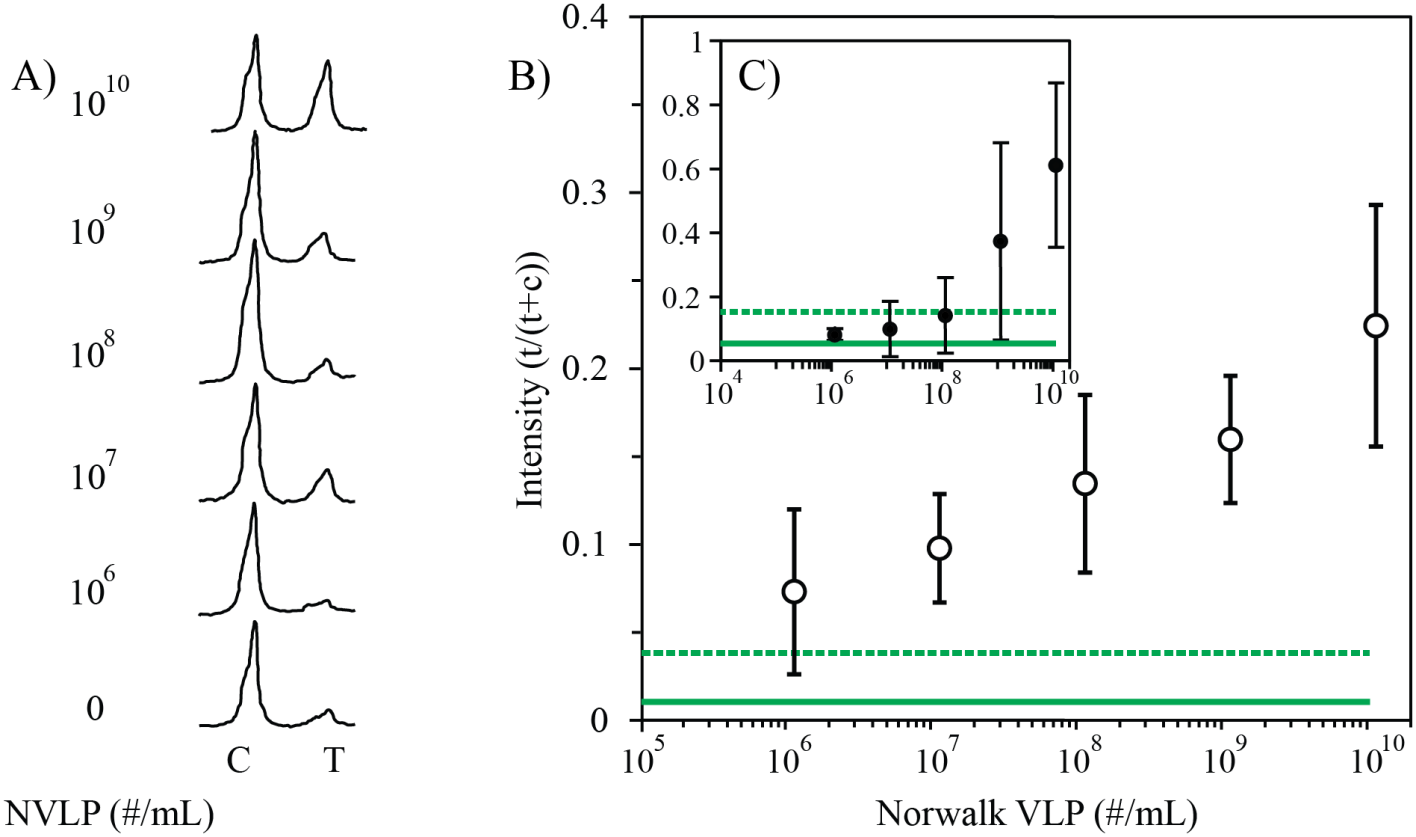
Evaluation of the Norwalk VLP (NVLP) phage LFA. A) Intensity plots of representative lateral-flow assay strips detecting Norwalk VLPs in 100 μL PBS, using phage-antibody construct and HRP/anti-M13 antibody conjugate, made in ImageJ software. These intensity plots were analyzed using the gel analysis tool in ImageJ. First peak is for the control line (C) and the second peak is for the test line (T). B) Intensity vs. NVLP concentration in sample for phage LFA (n = 5 or 6, average ± 1 SD). C) Intensity vs. NVLP concentration in sample for gold nanoparticle LFA (n = 3; average ± 1 SD). For B) and C), the intensity of the test line divided by the sum of the intensities of the test and the control lines of each strip were calculated for each strip. The solid lines represent the average value for the no-target control and the dotted lines represent the no-target value plus three times the standard deviation of the background.

To further analyze the strips, avoid subjectivity and confirm visual limit of detection, ImageJ’s gel analysis tool [42] was used to extract the line intensities from the images of the scanned strips from five independent experiments using two different batches of phage reporters; a total of six replicates for each concentration (intensity plots; Fig 4A). The area under each peak was then numerically integrated using the ImageJ Gel Analysis Toolbox, and the average intensity of the test line divided by the sum of the intensity of the control line and test line for each strip was plotted against the number of VLPs offered per strip for the phage LFA in Fig 4B and for the gold nanoparticle LFA in Fig 4C. Test line intensities increased with number of VLPs offered. The solid line represents the average intensity with no VLPs added, and the dotted line represents the no-target average plus three times the standard deviation of this background. The LoD, defined as the lowest concentration tested, at which the signal is clearly distinguishable from the no-target sample signal was estimated to be 10^7^ VLP/mL. It was also confirmed using t-test that signal for 1.15x10^7^ VLPs/mL and above was significantly different from the no-target control sample (S1 Table). For comparison, several immunochromatographic assays for noroviruses have been compared to RT-PCR as a gold standard [20], and the commercial, FDA-cleared ELISA tests for various genotypes report limits of detection from 5*10^7^–4*10^11^ viruses/mL [44]. LoDs are highly dependent not only on the antibody pair used and the genotype of the norovirus, but also on the sample matrix and sample quality and storage [14]. However, the phage LFA described in this study showed more than 100-fold lower limit of detection compared to the gold nanoparticle LFA using the same antibody sandwich pair.

## Conclusions

Noroviruses commonly are responsible for rapid gastrointestinal disease outbreaks in environments such as military vessels, cruise ships, hospitals, care centers, etc. There is a need for a simple point-of-care detection method which could be used to identify the source as well as carriers of the disease.

We have developed a sensitive lateral-flow assay for the detection of Norwalk virus-like particles, improving the limit of detection one hundred-fold compared to a conventional gold nanoparticle LFA using the same antibody sandwich pair. This is a first demonstration of the detection of VLPs in lateral-flow assay using phage nanoparticles as reporters and promises a more sensitive point-of-care detection method that would allow the prompt identification of the cause of symptoms and the application of infection control measures in a timely manner. This approach should in the future be tested with live virus and with additional antibodies for broader coverage, and could lead to a sensitive, convenient diagnostic test for Norwalk infection.

## Supporting Information

S1 Table Comparison of the no-target control to positive LFAs using t-test. (DOCX)
