# Supplementary material for: Sensitive Detection of Norovirus Using Phage Nanoparticle Reporters in Lateral-Flow Assay
Source: PLoS One. 2015 May 15;10(5):e0126571. doi: 10.1371/journal.pone.0126571 (PMC4433186; doi:10.1371/journal.pone.0126571)
Supplement: S1 Table — (DOCX) [file pone.0126571.s001.docx]

**S1 Table. Comparison of the no-target control to positive LFAs using t-test**

| Norwalk VLP/mL | 0 | 1.15x10^6^ | 1.15x10^7^ | 1.15x10^8^ | 1.15x10^9^ | 1.15x10^10^ |
| --- | --- | --- | --- | --- | --- | --- |
| Average of t/(t+c), $\bar{x}$ | 0.015 | 0.073 | 0.098 | 0.135 | 0.160 | 0.224 |
| St. Dev. of t/(t+c), σ | 0.012 | 0.047 | 0.031 | 0.050 | 0.036 | 0.069 |
| No of replicates | 5 | 6 | 6 | 5 | 6 | 6 |
| Variance of t/(t+c), s^2^ | 1.5x10^-4^ | 2.2x10^-3^ | 9.5x10^-4^ | 2.5x10^-3^ | 1.3x10^-3^ | 4.7x10^-3^ |
| Pooled variance, $s_{p}^{2}$ |  | 2.7x10^-6^ | 5.1x10^-7^ | 3.3x10^-6^ | 9.6x10^-7^ | 1.2x10^-5^ |
| t-test statistic, t_s_ |  | 2.061 | 4.457 | 3.762 | 6.635 | 5.290 |
| t_0.05/9_ = 2.262 |  | H_0_ | H_a_ | H_a_ | H_a_ | H_a_ |

The t-test statistic *t_s_* is defined as

$t_{s}=\frac{{\bar{x}_{po}- \bar{x}}_{ne}}{\sqrt{\frac{s_{p}^{2}}{n_{ne}}+\frac{s_{p}^{2}}{n_{po}}}}$ Eq.1

where, $s_{p}^{2}$is the pooled variance, $\bar{x}_{po}$ is the average t/(t+c) value for the positive control, $\bar{x}_{ne}$is the average t/(t+c) value for the no-target control, $s_{po}^{2}$ is the variance of the t/(t+c) value for the positive sample and $s_{ne}^{2}$ is the variance of the t/(t+c) value for the no-target control.

$s_{p}^{2}=\frac{\left( n_{po}-1 \right)s_{po}^{2}+\left( n_{ne}-1 \right)s_{ne}^{2}}{n_{po}+n_{ne}-2}$ Eq. 2

Six LFA replicates (or 5 in one case) for each concentration of Norwalk VLPs and five replicates for the no-target control were performed. The intensity of the test line (t) was divided by the sum of the intensities of the test and the control lines (t+c) of each strip and the average of the 5 or 6 replicates was calculated.

We took as the null hypothesis (H_0_) that there was no difference between the no-target control and the positive sample (significance level of α=0.05). Then the reject t-value of t_0.05/9_ was 2.262 for 9 degrees of freedom (n_no-control_ + n_positive_ - 2 = 9, n_no-target_: number of no-target replicate experiments and n_positive_: number of replicate experiments for positive samples).

We strongly reject the null hypothesis for all values 1.15x10^7^ VLPs/mL and above (t-test statistic > t_0.05/9_). For 1.15x10^8^ the number of the replicate experiments was 5 and thus t_0.05/8_ = 2.230 was used.
